# miR-29a inhibition normalizes HuR over-expression and aberrant AU-rich mRNA stability in invasive cancer

**DOI:** 10.1002/path.4178

**Published:** 2013-03-21

**Authors:** Wijdan Al-Ahmadi, Maha Al-Ghamdi, Norah Al-Souhibani, Khalid SA Khabar

**Affiliations:** Molecular Biomedicine Programme, King Faisal Specialist Hospital and Research CentreRiyadh, Saudi Arabia

**Keywords:** cancer invasion, breast cancer, post-transcriptional control, miRNA, mRNA stability, AU-rich elements

## Abstract

The activities of RNA-binding proteins are perturbed in several pathological conditions, including cancer. These proteins include tristetraprolin (TTP, *ZFP36*) and HuR (*ELAVL1*), which respectively promote the decay or stability of adenylate-uridylate-rich (AU-rich) mRNAs. Here, we demonstrated that increased stabilization and subsequent over-expression of *HuR* mRNA were coupled to TTP deficiency. These findings were observed in breast cancer cell lines with an invasive phenotype and were further confirmed in *ZFP36*-knockout mouse fibroblasts. We show that TTP–HuR imbalance correlated with increased expression of AU-rich element (ARE) mRNAs that code for cancer invasion genes. The microRNA miR-29a was abundant in invasive breast cancer cells when compared to non-tumourigenic cell types. When normal breast cells were treated with miR-29a, *HuR* mRNA and protein expression were up-regulated. MiR-29a recognized a seed target in the TTP 3′ UTR and a cell-permeable miR-29a inhibitor increased TTP activity towards HuR 3′ UTR. This led to *HuR* mRNA destabilization and restoration of the aberrant TTP–HuR axis. Subsequently, the cancer invasion factors uPA, MMP-1 and MMP-13, and cell invasiveness, were decreased. The *TTP*:*HuR* mRNA ratios were also perturbed in samples from invasive breast cancer patients when compared with normal tissues, and were associated with invasion gene expression. This study demonstrates that an aberrant ARE-mediated pathway in invasive cancer can be normalized by targeting the aberrant and functionally coupled TTP–HuR axis, indicating a potential therapeutic approach. Copyright © 2013 Pathological Society of Great Britain and Ireland. Published by John Wiley & Sons, Ltd.

## Introduction

Developing effective therapeutics for metastatic cancer requires an understanding of the complex molecular mechanisms, such as aberrant mRNA stability pathways, that underlie the invasive phenotype of tumour cells 1,2. Among common sequence features that affect mRNA stability are the AU-rich elements (AREs), which are present in nearly 4000 human transcripts 3, including labile mRNAs that participate in many transient processes. Sustained mRNA stabilization leads to over-production of growth factors and other mediators that participate in cancer processes. This stabilization can be caused by reduced activity of mRNA decay-promoting proteins, such as tristetraprolin (TTP, *ZFP36*), or increased activity of mRNA-stabilizing factors, such as human antigen R (HuR, *ELAVL1*) as reviewed recently 4–8. Our work aims to elucidate the details of the aberrant roles of these RNA-binding proteins and the possibility of experimental restoration of their normal expression and function to reduce the invasiveness of cancer cells.

HuR over-expression and its increased cytoplasmic localization have been demonstrated in different tumours, including breast, colon, gastric, glioma, lung, ovary and prostate 9–15. On the other hand, TTP was under-expressed in breast and colon tumour cells 16,17. Previously, we found that TTP is deficient in the highly invasive cell line MDA-MB-231, when compared to non-invasive MCF-7 and normal MCF10A and MCF12A breast cell lines 1. We have therefore focused our efforts on understanding the details of potential coupling between TTP and HuR and subsequent ramifications on gene products involved in cancer invasiveness. Specifically, we examined the feasibility of normalizing these abnormalities by new agents, such as microRNA-(miRNA)-inhibiting modalities.

miRNAs are a class of non-coding RNA molecules that serve as post-transcriptional regulators by inducing mRNA decay or inhibiting translation, and they contribute to the pathogenesis of many human malignancies 18. One miRNA is miR-29a, which is differentially regulated in various types of cancer and has tissue-specific functions 19–21. In this study, we show that a cell-permeable miR29a inhibitor was able to normalize aberrant mRNA stability in invasive breast cancer cells by reducing the levels of HuR in a manner that was dependent on TTP. This, in turn, led to a reduction in uPA, MMP-1 and MMP-13. This normalization of mRNA stability, via restoration of a proper TTP–HuR balance, reduced the invasiveness of breast cancer cells, suggesting a potential therapeutic approach.

## Materials and methods

### Cell lines

Breast cancer cell lines MDA-MB-231 and MCF-7, the normal-like breast MCF12A cell line, MCF10A cells and the HEK293 kidney cell line were obtained from ATCC (Rockville, MD, USA). MDA-MB-231, MCF-7 and HEK293 cells were cultured in Dulbecco's modified Eagle's medium (DMEM; Invitrogen, Carlsbad, CA, USA). MCF12A and MCF10A cells were cultured in Ham's F12–DMEM mixture and supplemented with 20 ng/ml epidermal growth factor (EGF), 0.01 mg/ml bovine insulin and 500 ng/ml hydrocortisone (Sigma, St. Louis, MO, USA). *TTP/zfp36*^+/+^ and *TTP/zfp36*^−/−^ mouse embryonic fibroblasts obtained from Dr Perry J Blackshear (NIH) have been described previously 22; these were grown in DMEM. All culture media were supplemented with 10% fetal bovine serum (FBS) and antibiotics.

### mRNA half-life and quantitative RT–PCR

Cells were cultured in six-well plates and either treated or untreated for 48 h with 50 nm miR-29a inhibitor as cell-permeable peptide-linked nucleotides [PNA; RRRQRRKKR-OO-ATTTCAGATGGTGCT (Panagene, Korea) or a scrambled control PNA sequence (here called miR-29a control inhibitor: RRRQRRKKR-OO-ATTAATGTCGGACAA)]. Actinomycin D (ActD) at 5 µg/ml was then used to block transcription. Total RNA was extracted at intervals, using Trizol (Sigma). The reverse transcriptase (RT) reaction was performed using SuperScript II (Invitrogen). Quantitative PCR (qPCR) was performed as multiplex reactions in a Chromo 4 cycler (Bio-Rad, Hercules, CA, USA), using FAM-labelled TaqMan probes (Applied Biosystems, Foster City, CA, USA) for TTP (*ZFP36*), HuR (*ELAVL1)*, uPA (*PLAU*), MMP-1 and MMP-13, while a VIC-labelled *GAPDH* probe was used as the endogenous control. *HuR* mRNA in TTP/zfp36^+/+^ and TTP/zfp36^−/−^ MEFs was quantified using FAM-labelled mouse *HuR* and normalized to VIC-labelled mouse *β-actin*. Samples were amplified in triplicate and quantification of relative expression was performed using the *ΔΔC*t method. The mRNA half-life determinations were deduced using one-phase exponential decay model described previously in detail 23. The specific TaqMan microRNA assay (Applied Biosystems) for miR-29a was used to measure miR-29a levels and normalized to the control RNU48, according to the manufacturer's instructions.

### Western blotting

Western blotting was performed as described previously 1. Primary antibodies were goat anti-TTP (1:200; Santa Cruz Biotechnology, Santa Cruz, CA, USA), goat anti-HuR (1:500; Santa Cruz), goat anti-uPA (1:200; Santa Cruz), mouse anti-*α*-tubulin (1:500), *β*-actin or goat anti-GAPDH–HRP (1:4000; Abcam, Cambridge, UK).

### miR-29a mimic and inhibitor experiments

The miR-29a mimic experiments were run by seeding MCF-10A cells in six-well plates at a density of 0.75 × 10^6^ and incubating overnight. Cells were then transfected for 48 h with 50 nm miR-29a pre-miR precursor (Applied Biosystems; this is a small partially double-stranded RNA that mimics miR-29a endogenous precursor miRNA, with the following mature sequence: UAGCACCAUCUGAAAUCGGUUA) or a negative control using Lipofectamine 2000 (Invitrogen). RNA and total protein extractions were performed for qRT–PCR and western blotting, respectively. For miR-29a inhibitor experiments, MDA-MB-231 cells were treated with 50 nm control inhibitor or miR-29 inhibitor for 48 h, followed by qRT–PCR, western blotting or invasion experiments. Transfection efficiency was monitored using fluorescent-labelled non-specific mimic or inhibitor.

### RNA immunoprecipitation

MDA-MB-231 or HEK293 cells were seeded in 100 × 20 mm culture dishes at a density of 3.6 × 10^6^ cells and incubated overnight. HEK293 cells, which are suitable for over-expression studies due to their high transfection efficiency and low TTP background, were transfected with expression plasmid (4.5 µg) for TTP, C124R or PCR3.1 control plasmid for 24 h. The RNA-IP procedure was described previously 1. The associated mRNA was subjected to qRT–PCR, as described above, using FAM-labelled probes for *HuR* and normalized to a VIC-labelled probe for *GAPDH* (Applied Biosystems). The small amount of *GAPDH* mRNA that co-precipitated with TTP protein, due to weak non-specific binding, was used as a normalization control 24,25. A similar experiment was performed using non-transfected MDA-MB-231 cells seeded at the same density and treated with miR-29a inhibitor or control for 48 h.

### RNA interference

RNA interference studies were performed using siRNA duplexes designed for silencing of *HuR* (NM_001419, sense 5′-GCCUGUUCAGCAGCAUUGG-3′, antisense 5′-CCAAUGCUGCUGAACAGGC-3′) and a control scrambled siRNA. All siRNAs, including non-specific controls, were custom-made by Metabion (Germany). The efficiency of siRNA silencing was determined by RT–PCR and western blotting.

### Plasmids, 3′ UTR constructs and deletion mutants

The reporter expression vectors were previously constructed by our group for HuR 3′ UTR 26 and TTP 3′ UTR 27; the control stable 3′ UTR was from the bovine growth hormone gene. The *TTP* and *C124R* mutant TTP constructs were kindly provided by Dr PJ Blackshear (NIH). The deletion TTP 3′ UTR cassettes were constructed using PCR. The miR-29a deletion mutant included a 3′ UTR region at 1040–1424 nt, while the miR-29a-proficient but ARE-deleted construct comprised a 3′ UTR region at 1040–1502. Briefly, the forward primer consisted of a region upstream of the RPS30 promoter, while the reverse primers were as follows: 5′-TGCGATGCAATTTCCTCATTTTATTCATAGATAGGAGACACTGGAACCTCA-3′ and 5′-TGCGATGCAATTTCCTCATTTTATTCATAGATAGGCAACGGCTTTGGCTAC-3′ for miR29a deletion and ARE deletion mutants, respectively. These constructs, generated by cloning-free PCR 28, contained the full expression cassettes, including the promoter, reporter and polyA signal.

### Reporter assays

HEK293 cells were incubated in 96-well clear-bottomed black plates (Matrix Technologies, Hudson, NH, USA) at a density of 3 × 10^4^ cells/well and incubated overnight. The cells were then transfected with 75 ng RPS30–SGFP–control 3′ UTR or RPS30–SGFP–TTP 3′ UTR reporter plasmids or 100 ng miR-29a deletion mutant PCR products, using Lipofectamine 2000. The following day, cells were treated with 50 nm miR-29a mimic control or miR-29a mimic for 24 h and fluorescence measured using BD Pathway 435 imager (BD Biosciences, San Jose, CA, USA). Fluorescence quantification was facilitated by ProXcell software 28. Data are presented as mean ± standard error of the mean (SEM) of total fluorescence intensity with replicate readings (*n =* 3–4).

A similar experiment was performed by transfecting cells with a RPS30 luciferase construct fused to HuR 3′ UTR and treating them with 50 nm miR-29a inhibitor or a control inhibitor for 48 h; 25 µl luciferase lysis buffer (Promega, Madison, WI, USA)/well was added. After 15 min, luciferase activity was quantified using the luciferase assay system (Promega) and a ZENYTH 3100 reader (Anthos Labtec).

### Confocal microscopy

MDA-MB-231 cells were seeded on coverslips at a density of 0.6 × 10^6^ in six-well plates, mounted on slides and incubated overnight (50% of the total well area). After 24 h, the cells were treated with 50 nm miR-29a inhibitor or control inhibitor for 48 h. The slides were formaldehyde-fixed, permeabilized with 0.05% Triton, treated with 1/500 primary antibody to HuR or 1:250 TTP and then with FITC-conjugated labelled secondary antibody (1:1000; 1 h at 37 °C). For F-actin experiments, MDA-MB-231 cells were treated with miR-29a inhibitor or control for 48 h and stained with phalloidin for visualization by confocal microscopy.

### Invasion assays

MDA-MB-231 cells were transfected with 0.25 µg luciferase PCR expression products and co-transfected with HuR siRNA or control siRNA, or were treated with miR-29a inhibitor or control PNA for 48 h. The cells were reseeded onto the upper chambers of 24-well invasion inserts of 8 µm pore membranes (BD Biosciences) in serum-free DMEM at a density of 3 × 10^5^ cells/well. The chemoattractant consisted of 600 µl 10% FBS added to the lower chambers and the cells were incubated for 24 h. The membranes were removed from the inserts and incubated with lysis buffer for 15 min. Luciferase was assessed using a kit (Promega) and a ZENYTH 3100 reader (Anthos Labtec).

### Breast cancer patient data and analysis

Clinically annotated data for 503 invasive breast cancer tumour samples and corresponding matched normal samples from 61 patients were obtained from The Cancer Genome Atlas (TCGA). This public database (https://tcga-data.nci.nih.gov/tcga/tcgaAbout.jsp) was established by the NCI and NHGRI and has declared the following: 'All samples in TCGA have been collected and utilized following strict human subjects protection guidelines, informed consent and IRB review of protocols'. We used the open access patient-unidentifiable data tier and mined through the Oncomine web portal (www.oncomine.com). Normalized gene expression – level 2 data (which are normalized signals per probe or probe set) – were downloaded along with clinical data, including type of breast cancer. The *TTP*:*HuR* mRNA ratios and their changes were assessed between matched normal breast tissues and tumour tissues. The log_2_ median-centred intensity ratios were used and converted to anti-log for the *TTP*:*HuR* mRNA ratios. The *t*-test with Welch's correction was used for comparison. Pairwise correlations (Spearman's test) were used to correlate the *TTPI*:*HuR* mRNA ratios with the gene-specific log_2_ median intensity ratios.

### Statistical analysis

Two-sample Student's *t*-tests were used to determine the significance of the difference when comparing two data columns. In addition, one-way ANOVA was used when there were three or more data columns, such as among the four different cell lines. Two-way ANOVA was used when comparing multiple sets of data that reflect the effect of two independent factors.

## Results

### TTP and HuR are highly deregulated in invasive breast cancer cells

**Figure 1:**
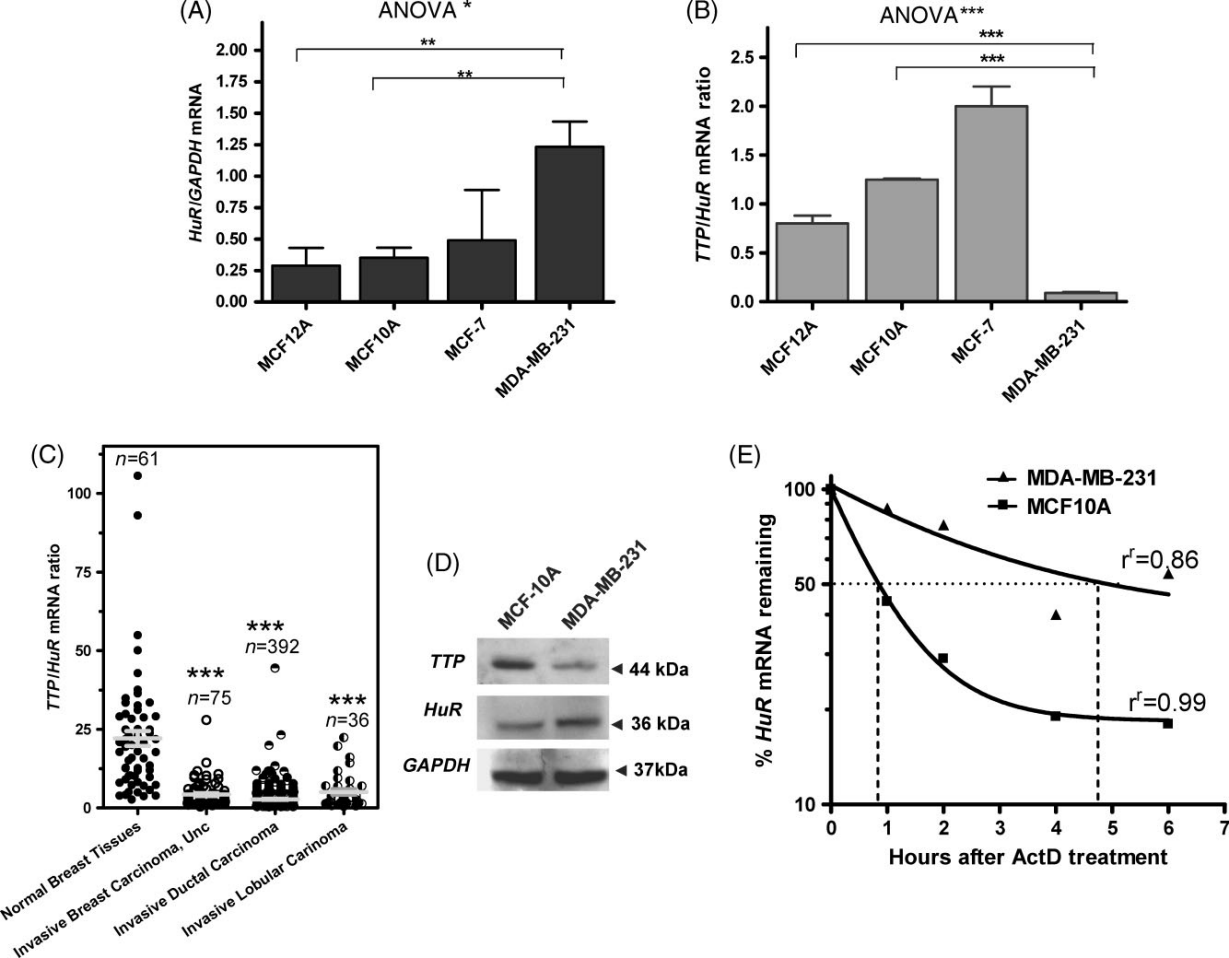
TTP:HuR ratio in invasive breast cancer. (A) *HuR* mRNA expression profile in normal and tumour breast cell lines. TaqMan assays for human *HuR* were performed and normalized to human *GAPDH*: values are mean ± SEM from three independent experiments; *p < 0.05 (one-way ANOVA), **p < 0.005 (Student′s *t*-test). (B) *TTP*:*HuR* mRNA expression ratio. RNA samples in (A) were also used for quantification of *TTP*, using TaqMan and normalized to human *GAPDH*. The ratio of *TTP* to *HuR* mRNA was then calculated; ***p < 0.001 (Student′s *t*-test). One-way ANOVA was also computed. (C) Comparison of *TTP*:*HuR* mRNA expression ratio of different breast carcinoma samples versus normal matched breast tissue obtained from TCGA data, using the Oncomine portal; ***p < 0001 (Student′s *t*-test) in comparison with normal sample values; Unc, not classified. (D) TTP and HuR protein levels in MCF10A and MDA-MB-231 cells. (E) *HuR* mRNA decay curve in MDA-MB-231 and MCF10A cells. The mRNA half-lives were calculated using the one-phase exponential decay model. The data are from one experiment, representative of three independent experiments; *r*^2^, goodness of fit.

We first evaluated the balance between *TTP* and *HuR* mRNA levels in several breast cancer cell lines: MCF12A and MCF10A cells representing normal-like breast cells, the MCF-7 tumour breast line and MDA-MB-231, a highly invasive cell line. The highest *HuR* mRNA expression was seen in MDA-MB-231 cells, 2.5-fold increased when compared to MCF-7 cells and five-fold greater than normal-like breast cells (Figure 1A). *TTP* mRNA expression was lowest in MDA-MB-231 cells, resulting in a very low *TTP*:*HuR* mRNA ratio (Figure 1B), almost 22-fold lower in MDA-MB-231 than in normal cell lines and the non-invasive MCF-7 line, which has a ratio of roughly 1.0. This expression pattern was confirmed by clinical data from TCGA database, using the Oncomine portal. Clearly aberrant *TTP*:*HuR* ratios were evident in invasive breast, invasive ductal and invasive lobular carcinomas, when compared to paired normal breast tissues (Figure 1C). At the protein level, HuR expression was higher while TTP was lower in the invasive MDA-MB-231 cell line than in MCF10A cells (Figure 1D). Increased *HuR* mRNA stability was also prominent in MDA-MB-231 cells (mRNA half-life > 4 h) when compared with the normal MCF10A cell line (mRNA half-life of nearly 1 h) (Figure 1E).

### TTP binds and regulates *HuR* mRNA

**Figure 2:**
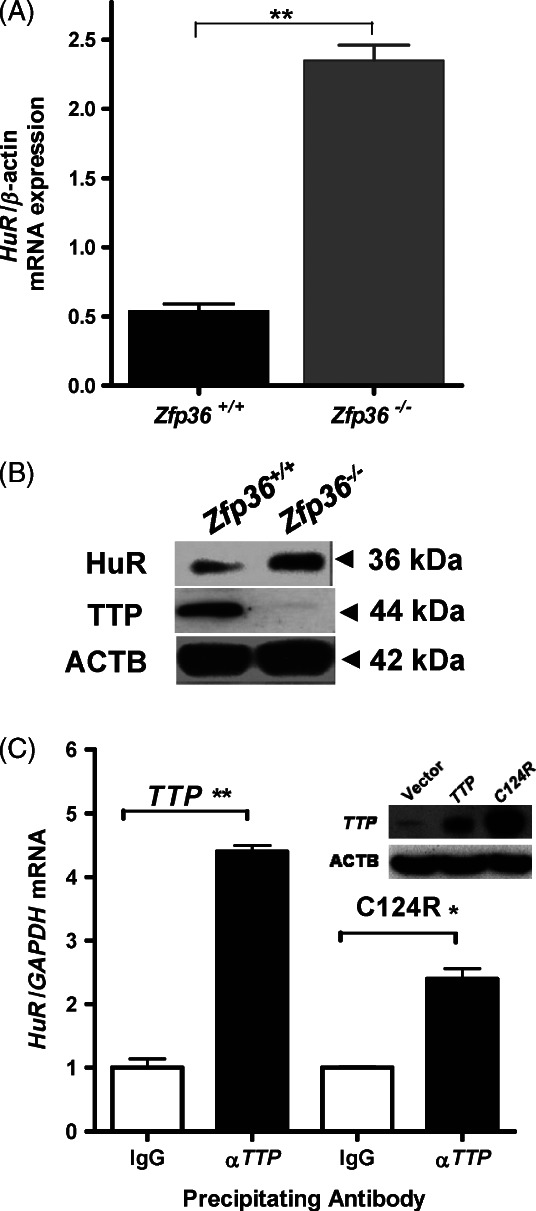
TTP regulation of HuR. (A) *HuR* mRNA expression in *Zfp36*^+/+^ and *Zfp36*^−/−^ MEFs. TaqMan for mouse HuR was performed and normalized to *β*-actin: values are mean ± SEM from three independent experiments; **p < 0.005 (Student's *t*-test). (B) HuR and TTP protein expression in *Zfp36*^+/+^ and *Zfp36*^−/−^ mouse fibroblasts, using western blotting. (C) Real-time PCR quantification of *HuR* mRNA associated with TTP protein. HEK293 cells were transfected with *TTP* or mutant *TTP* (C124R) expression plasmids, lysed and immunoprecipitated using anti-TTP or normal IgG control antibody. Quantification of physically associated *HuR* mRNA was performed by qPCR; *p < 0.05, **p < 0.005 (Student's *t*-test); (inset) western blotting of TTP and C124R expression.

The highly deregulated pattern of TTP–HuR expression, both in cell lines and in patient samples, prompted us to investigate whether *HuR* mRNA itself is a target for TTP-mediated mRNA decay and whether TTP deficiency in breast cancer is causative for HuR over-expression. Examination of *HuR* mRNA and protein expression in TTP/*Zfp36^−/−^* mouse embryonic fibroblasts revealed a five-fold increase compared to TTP/*Zfp36^+/+^* cells, indicating that TTP regulates HuR mRNA (Figure 2A and B). We determined whether this regulation was a result of a direct physical association of TTP protein with *HuR* mRNA by over-expressing TTP or a non-binding mutant (C124R) in HEK293 cells. When TTP-associated mRNA was collected by RNA-IP, we found that *HuR* mRNA was associated with the anti-*TTP–TTP* complex − *HuR*/*GAPDH* mRNA relative abundance was 4.5-fold − when compared to the IgG control (normalized ratio of 1.0; Figure 2C). Regular abundance of *HuR* to *GAPDH* mRNA, ie without RNA-IP, was 0.3 in HEK293 cells (data not shown) and 0.5–1.0 in the breast cancer cells (Figure 1A). A weaker association was seen with the *TTP* non-binding mutant, *C124R*, despite its over-expression (Figure 2C).

### Mir29a increases HuR expression and reduces TTP

**Figure 3:**
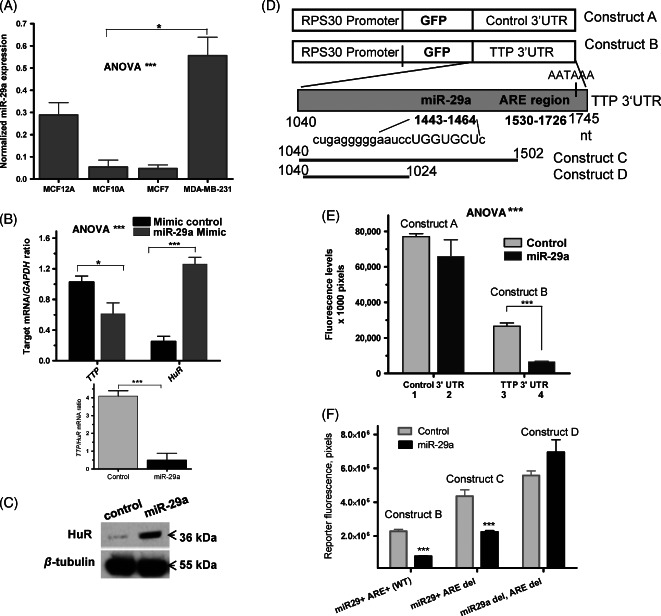
miR-29a levels and effects on TTP–HuR axis (A) miR-29a expression profile in normal and tumour breast cell lines. TaqMan expression assays for hsa-miR-29a were performed and normalized to RNU48 as the endogenous control: values represent mean ± SEM from three independent experiments; *p < 0.05 (Student′s *t*-test); ***p < 0.001 (one-way ANOVA). (B) Effect of miR-29a on *TTP* and *HuR* mRNA expression. MCF10A normal-like cells were transfected with miR-29a precursor hairpin or control for 24 h; qPCR for *TTP* and *HuR* was performed: ***p < 0.001 (two-way ANOVA); *p < 0.05, ***p < 0.001 (Student′s *t*-test); (Lower panel) *TTP*:*HuR* mRNA ratio. (C) Western blots for HuR and control *β*-tubulin proteins in MCF-10A cells with or without miR-29a (pre-miR) precursor. (D) Schematic representation of the RPS30-EGFP-control 3′ UTR and RPS30-EGFP-TTP 3′ UTR reporter constructs; arrow points to the regions of the TTP 3′ UTR deleted by cloning-free reporter construction. (E) Regulation of TTP 3′ UTR reporter expression by miR-29a mimic in HEK293 cells. GFP fluorescence was measured 24 h post-transfection; ***p < 0.001 (Student′s *t*-test). Two-way ANOVA is also shown. (F) HEK293 cells transfected with 100 ng expression constructs comprising either miR-29a site + ARE region, miR-29a site alone or miR-29a and ARE-deleted regions. The miR-29a mimic or mimic control (each at 12.5 nm concentration) was co-transfected with the indicated constructs and GFP fluorescence measured after 48 h; ***p < 0.0001 (Student′s *t*-test).

Next, we investigated the upstream mechanisms of TTP deficiency and their relationship to HuR over-expression by focusing on miR-29a, which has been reported to be highly expressed in metastatic RasXT cells relative to epithelial EpRas cells, and has been shown to suppress TTP 21. Increased expression of miR-29a was seen in the invasive cell line MDA-MB-231 compared to its normal-like counterpart MCF10A and non-invasive MCF-7 and MCF12A cells (Figure 3A). Furthermore, introduction of miR-29a in the form of a pre-miR precursor into normal MCF10A cells that lacked or expressed little miR-29a led to a 40% reduction in *TTP* mRNA, with a concomitant five-fold increase in *HuR* mRNA (Figure 3B, upper panel) and protein (Figure 3C) and a switch to an abnormally low TTP:HuR ratio (Figure 3B, lower panel).

We verified the effect of miR-29a on human TTP 3′ UTR by using an EGFP reporter construct containing TTP 3′ UTR that bears the seed target site for miR-29a. When we analysed the two miR target sequences reported by Gebeshuber *et al* 2009, they were mapped to the mouse mRNA of *TTP*/*ZFP36* (NM_011756). Only a single miR-29a binding site in the human *TTP/ZFP36* mRNA (NM_003407) was found and used in the construct, which was fused to a cellular non-inducible RPS30 promoter, which allows specific post-transcriptional assessment 29 (Figure 3D, *upper panel*). The reporters were co-transfected with a miR-29a precursor or a negative control into HEK293 cells. A four-fold reduction in TTP 3′ UTR reporter activity was seen as a result of miR-29a compared with the control (Figure 3E, columns 3 and 4), which demonstrated that *TTP* mRNA is targeted by miR-29a. Furthermore, the ARE-bearing 3′ UTR of TTP caused a significant reduction in GFP reporter activity compared to the control 3′ UTR, indicating the functionality of the TTP 3′ UTR (Figure 3E, columns 1 and 3; *p <* 0.001). We pinpointed that miR-29a indeed targets a sequence region and assessed the contribution of the ARE in the TTP 3′ UTR by creating mutants that had deletions of the miR-29a target region, the ARE region or both (Figure 3D, lower panel). miR-29a failed to exert its silencing activity when the miR-29a target sequence was deleted (Figure 3F). This silencing effect seems to be independent of the AREs found in the TTP 3′ UTR, although a slightly lower silencing was observed in ARE absence (Figure 3 F). These results are consistent with the observation that miR-29a over-expression contributes to the irregular TTP–HuR balance in breast cancer cells.

### Mir29a inhibitor normalizes aberrant TTP–HuR balance

**Figure 4:**
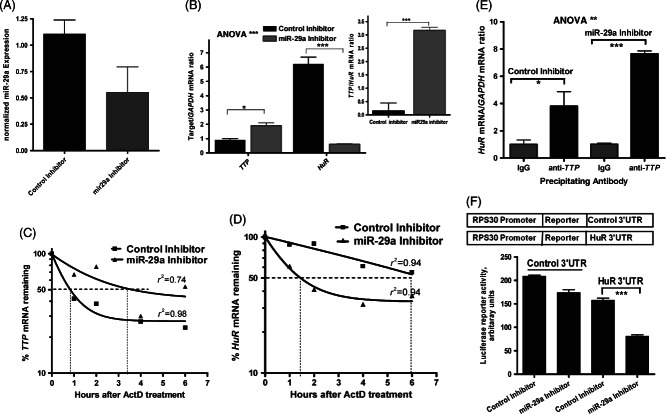
Normalization of TTP–HuR imbalance by the miR-29a inhibitor. (A) Knockdown efficiency of the cell-permeable miR-29a PNA inhibitor on miR-29a levels in MDA-MB-231breast cancer cells by qRT–PCR. (B) Effects of miR-29a inhibition on *TTP* and *HuR* mRNA expression in MDA-MB-231 cells by qRT–PCR; ***p < 0.001 (two-way ANOVA); *p < 0.05, ***p < 0.001 (Student′s *t*-test); (inset) *TTP*:*HuR* mRNA ratio for the control and miR-29a inhibited samples; ***p < 0.001 (Student′s *t*-test). (C, D) mRNA decay curves for *TTP* and *HuR* in MDA-MB-231 cells; curves, fitted using the one-phase exponential decay model, are from one experiment representative of at least two independent experiments; *r*^2^, goodness of fit. (E) Effect of miR-29a inhibition on *HuR* mRNA expression. MDA-MB-231 cells were treated with miR-29a inhibitor or control for 48 h, then TTP protein was immunoprecipitated with anti-TTP or IgG control antibody; *HuR* mRNA physically associated with TTP–anti-TTP complex was quantified by qPCR; **p < 0.01 (two-way ANOVA); *p < 0.05, ***p < 0.0001 (Student′s *t*-test). (F) Effect of miR-29a inhibition on HuR 3′ UTR luciferase reporter activity. MDA-MB-231 cells were treated with miR-29a inhibitor or control for 48 h, then transfected with an RPS30-Luc-HuR 3′ UTR reporter plasmid or control plasmid. Luciferase activity was measured after 24 h; ***p < 0.001 (Student's *t*-test).

In an attempt to substantiate that TTP–HuR imbalance can be restored, we down-regulated expression of miR-29a in MDA-MB-231 cells, using a cell-permeable miR-29a inhibitor. At 50 nm, an almost 50% reduction in miR-29a was achieved (Figure 4A). MiR-29a inhibition caused an increase in *TTP* mRNA of approximately two-fold (*p <* 0.01), with a parallel reduction in *HuR* mRNA levels (> four-fold; Figure 4B) and the ratio of *TTP*:*HuR* mRNA in the presence of miR-29a inhibitor was nearly 15 times greater than without inhibition (Figure 4B, inset). This TTP–HuR switch was also reflected at the mRNA stability level. The half-life of *TTP* mRNA (∼1 h) was increased nearly three-fold, while *HuR* mRNA became destabilized four-fold, from approximately 6 h to 1.5 h half-life (Figure 4C, D). These results indicate that inhibition of miR-29a can restore a closer-to-normal TTP–HuR balance by triggering selective ARE mRNA destabilization in invasive breast cancer cells. Further confirmation was provided by quantification of *HuR* mRNA that was physically associated with TTP protein, where increased binding of TTP to *HuR* mRNA occurred during miRNA inhibition (Figure 4E). When HuR 3′ UTR was fused to a luciferase reporter and transfected into MDA-MB-231 cells, followed by treatment with miR-29a inhibitor or control, the inhibitor decreased luciferase activity due to HuR 3′ UTR by two-fold (Figure 4F). These data indicate that miR-29a inhibition increases TTP activity towards HuR 3′ UTR.

### Mir29a inhibitor reduces HuR protein and invasiveness of breast cancer cells

**Figure 5:**
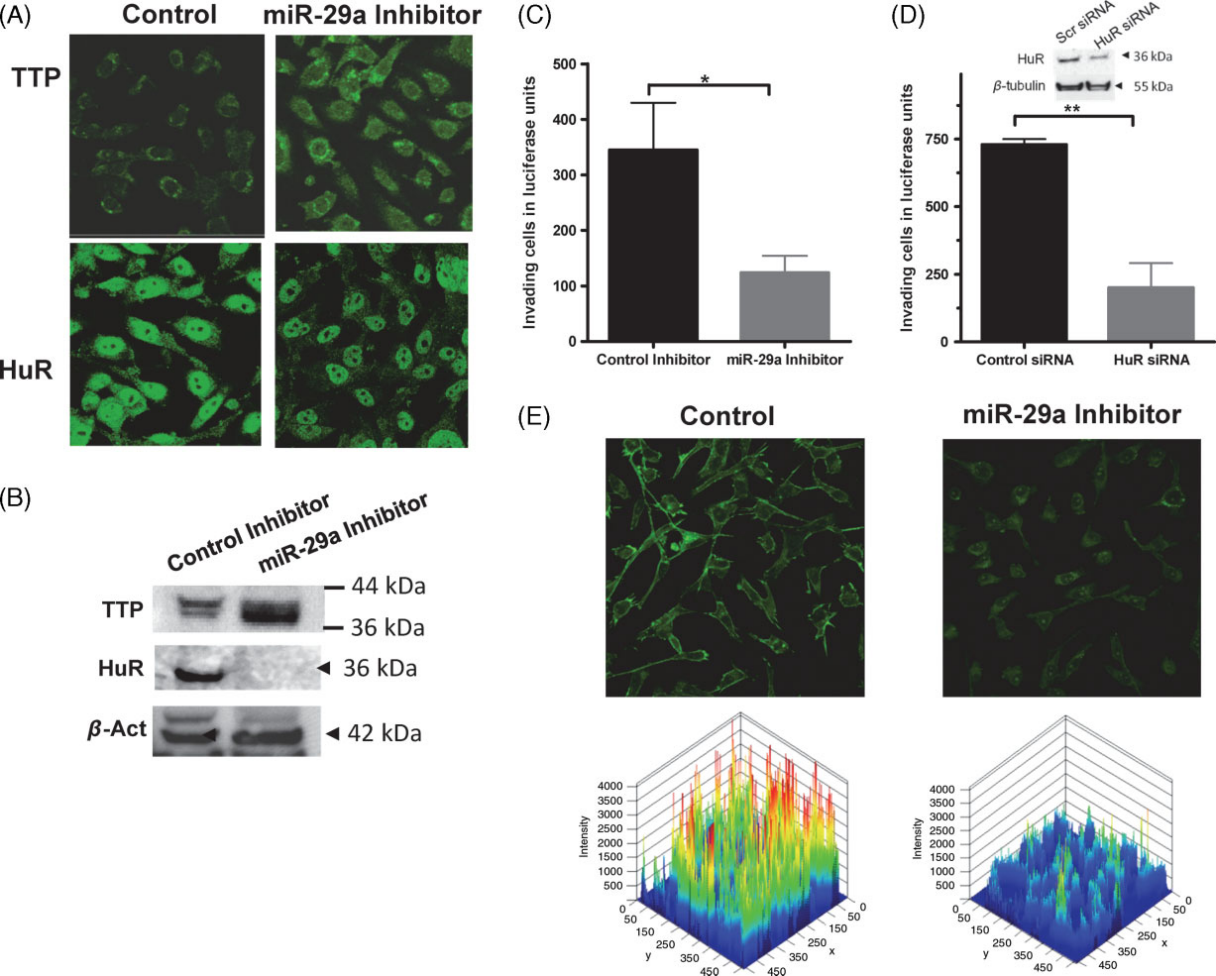
Phenotypic changes and invasion of invasive breast cancer cells following miR-29a inhibition. (A) Confocal microscopy of TTP and HuR protein as a result of miR-29a inhibition. (B) Western blotting using antibodies against TTP, HuR and *β*-actin as a loading control. (C, D) Effects of miR-29a inhibition and HuR knockdown on invasiveness of breast cancer cells: (upper panel) representative western blot of HuR siRNA knockdown efficiency. (E) Effect of miR-29a inhibitor on F-actin polymerization in breast cancer cells detected with phalloidin: (lower panel) graphical representation of fluorescence intensity.

Confocal microscopy revealed a noticeable increase in TTP (which is largely cytoplasmic), with a corresponding decrease in HuR in response to treatment with miR-29a inhibitor (Figure 5A). There was strong reduction in cytoplasmic HuR due to miR-29a inhibition, and nuclear HuR was reduced to a lesser degree, possibly because the antibody staining in the nucleus is more condensed and saturated. Western blotting demonstrated a decrease in HuR protein to an almost undetectable level upon miR-29a inhibition (Figure 5B). When MDA-MB-231 cells were treated with miR-29a inhibitor, the invasive potential of the cells was reduced by 64% compared to the control (Figure 5C). Similarly, knockdown of HuR by a specific siRNA resulted in 72% reduction in invasion (Figure 5D; upper panel inset shows the efficiency of HuR knockdown). Confocal microscopy of miR-29a inhibitor-treated cells stained with phalloidin revealed a reduction in F-actin staining and different morphology of the cells, with fewer pseudopodial protrusions (Figure 5E, upper panel). The lower panel is a graphical representation of the reduction in F-actin fluorescence intensity due to miR-29a inhibitor treatment.

### miR-29a inhibition-mediated reversal of pro-invasion gene expression

**Figure 6:**
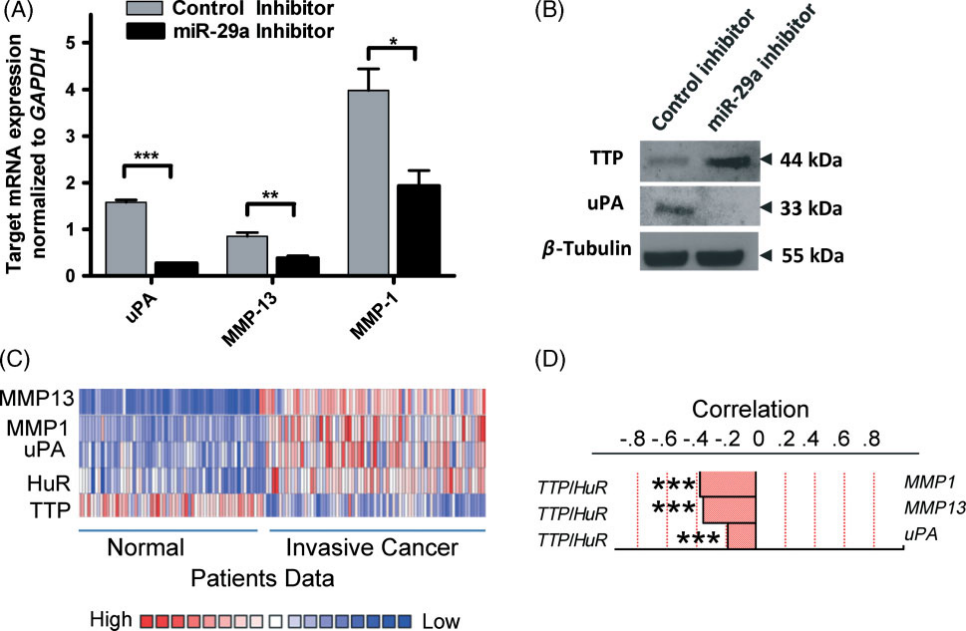
TTP–HuR balance modulation and pro-invasion/migration genes in cells and patient tissues. (A) mRNA expression for *uPA*, *MMP-1* and *MMP-13* pro-invasion factors in MDA-MB-231 cells treated with miR-29a inhibitor or control; ***p < 0.005 (Student's *t*-test). (B) Western blot of TTP and uPA protein expression normalized to *β*-tubulin endogenous loading control. (C) Heat map for TTP, HuR, uPA, MMP1 and MMP-13 in normal and invasive breast cancer patients, obtained from the TCGA database through the Oncomine web portal. (D) Graphical representation of pairwise correlation with *TTP*:*HuR* mRNA ratio in invasive breast cancer patients; level 2 gene expression data were obtained from TCGA data, using the Oncomine algorithm; ***p < 0.001, *p < 0.05 (Spearman's correlation).

**Figure 7:**
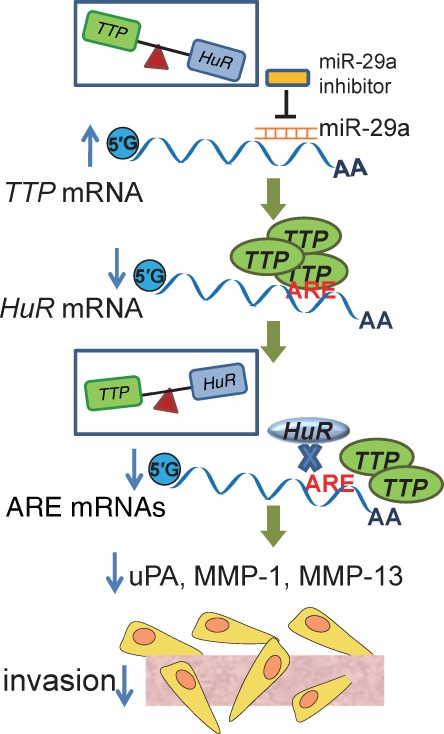
TTP–HuR pathway cascade following miR-29a inhibition. Inhibition of miR-29a relieves post-transcriptional repression of TTP; more TTP is produced that binds to and destabilizes *HuR* mRNA and other pro-cancer mRNAs. As a result, a normalized TTP–HuR balance is created, leading to enhanced ARE mRNA instability for factors involved in invasion and migration, such as *uPA*, *MMP-1* and *MMP-13* and a reduction in migration and invasion.

We examined the effect of the corrected TTP–HuR balance imparted by miR-29a inhibition on pro-invasion/ metastasis genes by examining *uPA*, *MMP-13* and *MMP-1* mRNAs (Figure 6A), which harbour AREs in their 3′ UTRs 1,3. As an example, a significant reduction in uPA protein levels was observed due to miR-29a inhibition (Figure 6B). These results demonstrate the importance of normalization of the TTP–HuR axis in regulating the major factors for invasion, and subsequently controlling the invasiveness and metastatic potential of breast cancer. The clinical information from TCGA was also examined and patterns of up-regulation were found for uPA, MMP-1 and MMP-13 (Figure 6C). Notably, the *TTP*:*HuR* ratio showed a statistically significant negative correlation with all of these invasion/migration mRNAs (Figure 6D), supporting the findings of this study. Figure 7 presents a graphical summary of the cascade of events supported by all of the findings.

## Discussion

Increased stabilization of mRNAs coding for key cancer genes can contribute to the tumourigenicity and maintenance of cancer cells. This can occur via increased expression and activity of the key stabilizing RNA-binding protein, HuR, or due to deficiency of TTP that promotes mRNA destabilization (reviewed in 4,8). Although the loss of TTP in breast cancer cells 1 and in other cell types, such as colon cancer 16,17, occurred with increased HuR expression 30–32, no causative link has been established between TTP deficiency and HuR over-expression. In the present study, we have demonstrated that *HuR* mRNA is associated with, and may be regulated by, TTP. Thus, TTP deficiency and HuR overexpression, observed both in the invasive breast cancer patient data presented here and in the highly invasive breast cancer cell line, MDA-MB-231, is a cause–effect relationship. More importantly, this study provides detailed experimental evidence to show that TTP–HuR imbalance and its subsequent downstream cascade can be normalized by a miR-29a inhibitor that alleviates miR-29a-mediated post-transcriptional repression of its target, TTP. The corrected TTP–HuR balance normalizes aberrant ARE mRNA stability for key gene products involved in invasion of breast cancer.

The TTP–HuR imbalance is caused largely by a TTP deficiency as well as by HuR over-expression. Reduction in TTP compared to normal tissues has been observed in a number of tumours, including thyroid, lung, ovary, uterus, cervix, brain, head and neck and breast 17,33–35. We previously found that TTP restoration in MDA-MB-231 cells promotes the decay of uPA and uPAR, resulting in a decrease in the invasive capacity of these cells 1. TTP also has multiple important targets, including COX-2, HIF-1*α*, IL-8, IL-6, IL-8, and VEGF 34,36–40 and the oncogenic Ser/Th kinase Pim-1, which is overexpressed in several cancers and promotes cellular growth and apoptosis resistance 41,42. Thus, TTP appears to be a down-regulator of many cancer-related genes, which substantiates its anti-tumour role. The demonstration of *HuR* mRNA as another target for TTP indicates that HuR may exacerbate invasive cancer processes, since it binds and stabilizes almost the same mRNA targets as TTP and auto-amplifies itself in an ARE-mediated fashion 43. The loss of TTP can lead to up-regulation of an array of *TTP*/*HuR* mRNA targets coding for proteins involved in maintenance of cellular growth, angiogenesis, invasion and metastasis. Analysis of TCGA breast cancer patient information supports the observations of aberrant TTP:HuR ratios, along with significant correlation with increased expression of invasion genes. This pattern of aberrant expression is similarly replicated in the well-established cellular model of invasive breast cancer used here, namely MDA-MB-231 cells 44. The cells are oestrogen-negative and have a basal B-subtype molecular signature 45. Our finding of a TTP–HuR imbalance and its correlation with the aberrant ARE mRNA expression in the cell line substantially mirrors the patient data.

Knowledge of the upstream causes for TTP deficiency can provide further insight into the TTP–HuR imbalance and its ramifications (Figure 7) and suggest possibilities for experimental correction and therapeutic targeting. Since miR-29a is over-expressed in mesenchymal metastatic RasXT cells compared to epithelial EpRas cells, and also in breast cancer tissues 21, we questioned whether inhibition of miR29a could correct the TTP–HuR imbalance. The cell-permeable peptide nucleic acid (PNA) miR-29a inhibitor shows high affinity, sequence specificity, stability and lower toxicity 46,47, while incorporation of this cell-permeable peptide avoids the need for transfection reagents. The miR-29a inhibitor down-regulated miR-29a levels and corrected the TTP–HuR imbalance by increasing *TTP* mRNA levels and subsequent *HuR* mRNA destabilization. This apparently occurred first by blocking miR-29a recognition to its target seed site in the TTP 3′ UTR, since the reporter with miR-29a target deletion failed to respond to miR-29a. The increase in TTP molecules available to target HuR 3′ UTR results in increased *HuR* mRNA recognition by TTP, as demonstrated in the RNA-IP experiments. The normalization of aberrant ARE mRNA-mediated pathway by miR-29a inhibition caused significant reduction in invasion of breast cancer cells. The latter activity was seen at different experimental levels, with reductions in actin polymerization, pseudopodia formation that assist metastasis 48, and invasion through Matrigel.

The imbalanced TTP–HuR axis clearly contributes to the progression of cancer, particularly invasion and metastasis, in the context of post-transcriptional regulation. Since mRNA stability cannot be assessed in patient tissues as it can in cell lines, measurement of TTP:HuR ratios –at either mRNA or protein levels – may be used as a reflection of mRNA stability changes. This concept may bring the present findings closer to use in diagnostic, prognostic or therapeutic monitoring of invasive breast cancer patients. The fact that the perturbed ARE-mediated pathway in invasive breast cancer cells can be normalized, at least in the laboratory, by use of a cell-permeable miR-29a inhibitor (Figure 7), suggests that aberrant RNA–protein interactions in disease can be targeted by nucleic acid therapeutics or small-molecule drugs. However, there is still a lack of these specific therapeutics that may have benefits in humans at this time. If further confirmed *in vivo*, triggering mRNA instability by inducing TTP and reducing HuR may be approached as a targeted therapy modality, not only in invasive breast cancer but also in other cancer types and diseases, such as auto-immune and chronic inflammatory states.
